# Hormonal and Hydroxycinnamic Acids Profiles in Banana Leaves in Response to Various Periods of Water Stress

**DOI:** 10.1155/2014/540962

**Published:** 2014-05-27

**Authors:** Jalel Mahouachi, María F. López-Climent, Aurelio Gómez-Cadenas

**Affiliations:** ^1^Departamento de Ingeniería, Producción y Economía Agraria, Carretera de Geneto 2, La Laguna, 38200 Santa Cruz de Tenerife, Spain; ^2^Departamento de Ciencias Agrarias y del Medio Natural, Universidad Jaume I, Campus Riu Sec, 12071 Castellón de la Plana, Spain

## Abstract

The pattern of change in the endogenous levels of several plant hormones and hydroxycinnamic acids in addition to growth and photosynthetic performance was investigated in banana plants (*Musa acuminata* cv. “Grand Nain”) subjected to various cycles of drought. Water stress was imposed by withholding irrigation for six periods with subsequent rehydration. Data showed an increase in abscisic acid (ABA) and indole-3-acetic acid (IAA) levels, a transient increase in salicylic acid (SA) concentration, and no changes in jasmonic acid (JA) after each period of drought. Moreover, the levels of ferulic (FA) and cinnamic acids (CA) were increased, and plant growth and leaf gas exchange parameters were decreased by drought conditions. Overall, data suggest an involvement of hormones and hydroxycinnamic acids in plant avoidance of tissue dehydration. The increase in IAA concentration might alleviate the senescence of survival leaves and maintained cell elongation, and the accumulation of FA and CA could play a key role as a mechanism of photoprotection through leaf folding, contributing to the effect of ABA on inducing stomatal closure. Data also suggest that the role of SA similarly to JA might be limited to a transient and rapid increase at the onset of the first period of stress.

## 1. Introduction

It is currently well known that phytohormones are involved in the regulation of numerous physiological processes including seed dormancy, plant development, and responses to biotic and abiotic stresses in many higher plants. Under drought conditions, the induction of ABA biosynthesis initiates the signaling pathways within plant tissues, leading to numerous molecular and cellular responses, such as the expression of stress related genes and stomatal closure [1–4]. In this regard, ABA plays a key role in root-to-shoot signaling under drought inducing adaptive responses [3, 5, 6]. In general, endogenous ABA increased under water deficit and was reestablished to normal levels immediately after stress release [7–9].

Jasmonic acid (JA) and derivatives named jasmonates are a group of naturally occurring plant growth regulators [10], considered as essential components of the signaling pathway triggering the expression of plant defense genes in response to various environmental stresses [11]. JA has been implicated in various physiological processes [12] such as plant-pathogen interactions [13], wounding [14], water deficit [9, 15, 16], and salt stress [17].

The involvement of IAA in plant stress responses is less known; however, it has been reported that initial water deficit strongly reduces IAA in the blade and increases it in the root of* Lupinus albus* [18]. An increase in IAA has been also observed in roots of citrus genotypes subjected to flooding stress [19]. In addition, exogenous application of IAA appears to alleviate the effects of drought in some plant species [20, 21].

Salicylic acid (SA) is involved in the regulation of various physiological processes in plants [22] such as growth, photosynthetic performance, ion uptake, and membrane permeability [23–25]. It is also considered a signal molecule that modulates plant responses to drought [26], salt stress [27–29], heavy metals [30], and multiple-stress tolerance [31].

On the other hand, hydroxycinnamic acids such as ferulic acid and cinnamic acid appear to be associated with plant abiotic stress responses. Ferulic acid seems to be the main emitter of the blue-green fluorescence of leaves and may act as a light fitter limiting mesophyll penetration under drought conditions and can also support drought adaptation by downregulation of leaf growth [32, 33]. Foliar cell-wall-bound ferulic acid levels increased as a response to water deficit, which could be one of the protective mechanisms induced by drought conditions [33]. Other cell-wall-bound metabolites such as cinnamic acid, caffeic acid, and p-coumaric acid, which are present in some plants in low amounts, contribute very little to the overall blue-green fluorescence emission of leaves. Plants which do not possess ferulic acid in their cell walls, such as sunflower, pumpkin, or tobacco, exhibit only slight blue-green fluorescence emission [32]. Cinnamic acid (CA) is one of the strongest allelochemicals [34] which can stimulate antioxidant enzyme activities under pathogen [35] and chilling stress [36].

Banana is a tropical crop which requires a high water supply because of its significant leaf area. Decreases of growth, transpiration rate, stomatal conductance, and photosynthetic rate were reported in banana plants subjected to water deficit [37–43]. However, it has been indicated that banana plants are able to maintain their internal water status during drought by reducing radiation load and closing stomata [40]. The natural folding of banana laminae even in well irrigated plants reduces photochemical damage by reducing the flux density of radiation intercepted by the lamina surface [41]; however, folding is greatly accentuated under the decline of water availability. Previously, we have reported that banana plants increased their leaf mineral concentration under water deficit to help maintain leaf relative water content because of osmotic adjustment mechanism [43].

Although plant hormones have been implicated in the responses of plants to water stress conditions, only the connection between ABA and the control of adaptive responses to drought stress has been extensively studied so far. In the present work, we investigated the pattern of change of several hormones and hydroxycinnamic acids in banana plants subjected to various periods of water stress in order to elucidate the involvement of these metabolites in the control of the physiological water stress responses under repetitive periods of drought.

## 2. Materials and Methods

### 2.1. Plant Material

Three-month-old banana (*Musa acuminata* AAA “Grand Nain”) plants were used to study the responses of banana to several periods of drought. “Grand Nain” is well adapted to subtropical conditions and was used previously for investigating banana water stress physiology [42, 43]. Plants received from the nursery were transplanted and grown in 50 l plastic pots (one plant per pot) containing peat substrate (Leader potting soil, Germany) under glasshouse conditions. Available nutrients in substrate were N (200 mg L^−1^), P_2_O_5_ (200 mg L^−1^), and K_2_O (300 mg L^−1^). Before transplanting, 50 g/pot of granular fertilizer (Osmocote Pro, NPK fertilizer containing Mg with trace elements: 18(N)-9(P)-10(K)-2Mg-Te) was incorporated into the substrate. Throughout the experimental period, plants were grown under conditions of 20–30°C of temperature, 70–90% of relative humidity, and 1200 *μ*mol m^−2^ s^−1^ of maximum photosynthetically active radiation (PAR).

### 2.2. Treatments of Water Stress

Thirty days after plant acclimation, plants were subjected to a series of water regimes. The duration of water stress (WS) periods increased progressively as follows: WS1 (9 days), WS2 (14 days), WS3 (25 days), WS4 (34 days), WS5 (45 days), and WS6 (57 days). After each period of drought, plants were rehydrated during at least two weeks for recovery. In contrast, control plants were successfully irrigated, three times a week.

### 2.3. Plant Growth and Sampling

At the end of each period of water stress, plant height, stem circumference, leaf area, and number of emerging leaves were determined. Leaf area (*S* (cm^2^)) was measured following the formula *S* = *L* × *l* × 0.8, where *L* is leaf blade length (cm) and *l* is leaf blade width (cm) according to Obiefuna and Ndubizu [44]. Samples of leaves, the second or third counting from plant apex, were harvested, frozen in liquid nitrogen, lyophilized, and stored at –20°C until analysis.

### 2.4. Soil Moisture

Volumetric soil moisture was recorded regularly at the established dates of water stress using a Trime-FM Time Domain Reflectometry (TDR) instrument (Imko equipment, Germany) as described previously [45]. In brief, the instrument was equipped with two-rod connector probes 15 cm in length and spaced by 5 cm. One permanent probe per pot was vertically inserted in the substrate at a depth of 15 cm. This equipment determines the percentage of volumetric soil moisture content.

### 2.5. Leaf Relative Water Content

Leaf relative water content (RWC) was determined at the end of each period of water stress using the third fully expanded leaves counting from the plant top. After sampling, leaf fresh weight (FW) was determined, and then leaves were hydrated until saturation in distilled water for 24 h at 4°C. Once surface dried, leaves were reweighed to obtain leaf turgid weights (TW). Subsequently, leaves were oven-dried at 70°C for 48 h and their dry weight (DW) was determined. Leaf RWC was calculated following the formula RWC (%) = (FW − DW)/(TW − DW)∗100.

### 2.6. Leaf Gas Exchange

Photosynthetic rate (*A*), stomatal conductance (gs), and transpiration rate (*E*) were determined regularly in banana leaves throughout the experimental period, using an LCpro portable photosynthesis system (ADC Bioscientific Ltd., Hoddesdon, UK) as described previously [9]. Determinations were performed on fully expanded leaves, generally, the third leaf counting from plant apex. Measurements were made in the morning (8:00 to 10:00 h); temperature within the leaf chamber was 25 ± 3°C and leaf-to-air vapor pressure deficit was 1.7 ± 0.3 KPa.

### 2.7. Determination of Hormonal and Hydroxycinnamic Acids

Plant hormones and hydroxycinnamic acids were analysed by liquid chromatography coupled to tandem mass spectrometry [46]. In brief, 25 *μ*L of a mixture of internal standards containing 5 ng of [^2^H_2_] IAA (Isotech, Sigma-Aldrich), 100 ng of [^2^H_6_] ABA, 100 ng of [^2^H_6_] JA, 100 ng of [^2^H_6_] SA (Isotech, Sigma-Aldrich, St. Louis, MO, USA), and 100 ng of [^2^H_6_] CA (Isotech, Sigma-Aldrich, St. Louis, MO, USA) was added to 0.05 g of powdered plant material. The tissue was homogenized in 5 mL of ultrapure water and extracts were then centrifuged at 5000 ×g for 10 min to pellet debris. The pH of the supernatant was adjusted to 2.8 with 15% CH_3_COOH and partitioned twice against an equal volume of diethyl ether. After discarding the aqueous phase, the organic fraction was evaporated in vacuum at room temperature and the solid residue resuspended in one mL of a water/methanol (90/10, v/v) solution which was filtered through a 0.22 *μ*m cellulose acetate filter. A 20 *μ*L aliquot of this solution was then directly injected in the HPLC system, a Waters-Milford, MA, USA, Alliance 2690 system coupled to a tandem mass spectrometer (geometry quadrupole-hexapole-quadrupole, Quatro LC, Micromass, Manchester, UK) through an orthogonal Z-spray electrospray interface. Concentrations of each plant hormone were determined using calibration curves performed with known amounts of pure standard samples.

### 2.8. Experimental Design and Statistical Analyses

Plants were distributed in three blocks with 36 plants each (18 control and 18 drought-stressed plants). Three plants per treatment and block were used for growth and gas exchange parameter measurements, and, at different dates (9, 14, 25, 34, 45, and 57 days after water stress period), three plants per treatment and block were randomly chosen and sampled to determine leaf RWC and hormone and hydroxycinnamic acids concentrations. In general, the third leaf was used for RWC determination and the second one counting from plant apex was used for hormone and hydroxycinnamic acids analysis. Mean values were compared using the least significant difference (LSD) test (*P* ≤ 0.05). Statistical analyses were performed using Systat 10 (SPSS Inc., Chicago, IL, USA).

## 3. Results 

### 3.1. Soil and Plant Water Status

**Figure 1:**
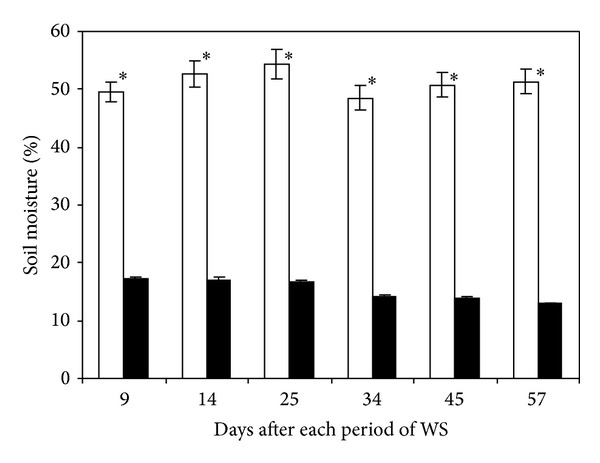
Soil moisture (%) in watered (□) and nonwatered pot soils (■) of banana plants. Data are means ± SE and each value was determined by three TDR probes with three replicates per treatment (*n* = 9) (one probe per pot). Data were compared for each date using the least significant difference (LSD) test. Significant differences at *P* ≤ 0.05 are represented by an asterisk (∗). WS: water stress.

Soil moisture was maintained between 49 and 54% in regularly irrigated plants during the experimental period. However, at the end of each period of drought soil moisture reached a minimum value (17–12%) related to the duration of water deprivation (Figure 1).

**Figure 2:**
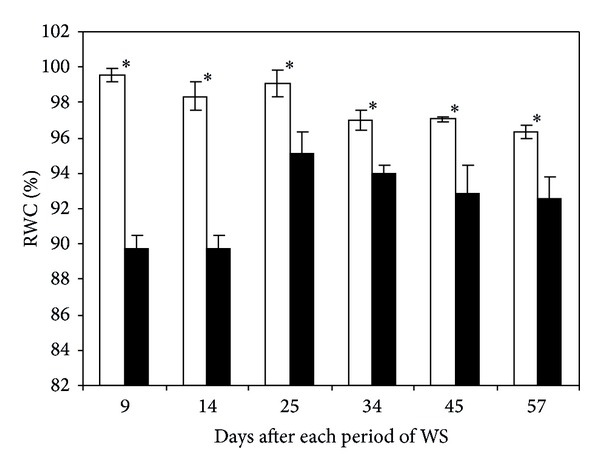
Leaf relative water content (%) in fully expanded leaves of banana plants. Treatments were regularly irrigated (□) and water-stressed (■) banana plants. Data are means ± SE and each value was determined in three different plants with three replicates per treatment (*n* = 9). Data were compared for each date using the least significant difference (LSD) test. Significant differences at *P* ≤ 0.05 are represented by an asterisk (∗). WS: water stress.

Leaf RWC oscillated between 96 and 99% in well irrigated plants throughout the whole trial period (Figure 2). The major reduction of RWC occurred at the end of the first two cycles of drought and decreases were about 10 and 9%, respectively, compared to control. Thereafter, RWC decrease determined after the various cycles of water stress was less severe and varied between 3 and 4% with respect to control.

### 3.2. Plant Growth

**Figure 3:**
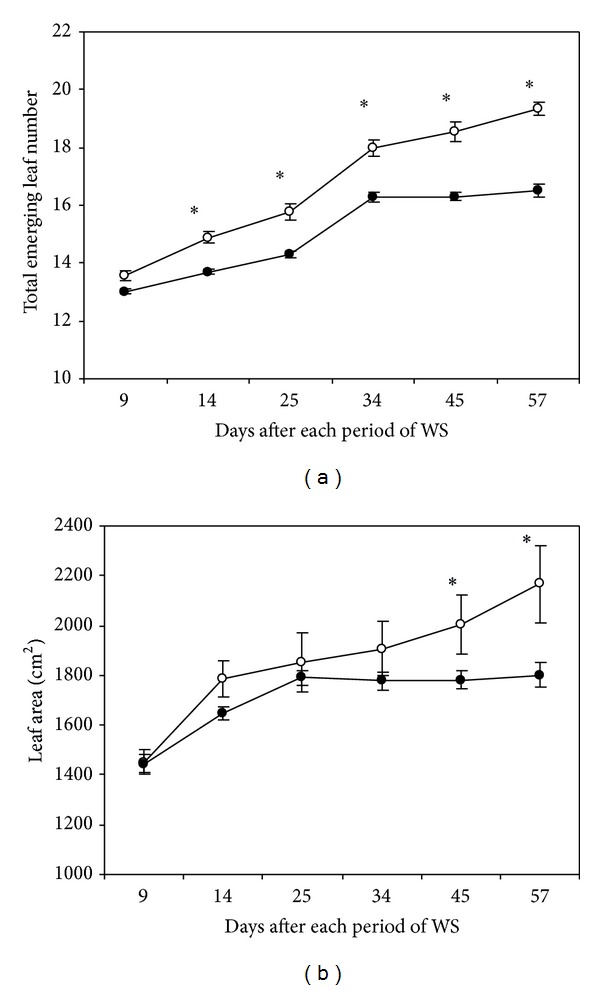
Total emerging leaf number (a) and leaf area (b) determined in regularly irrigated (○) and water-stressed (●) banana plants. Data are means ± SE and each value was determined in three different plants with three replicates per treatment (*n* = 9). Data were compared for each date using the least significant difference (LSD) test. Significant differences at *P* ≤ 0.05 are represented by an asterisk (∗). WS: water stress.

Leaf number in well irrigated plants increased from 13 to 19 leaves throughout the experimental period. Water stress clearly decreased leaf number since the 2nd (8%) until the last period of drought (15%) in comparison to control (Figure 3(a)). Meanwhile, leaf area increased continuously in watered plants; however, soil moisture exhaustion significantly detained leaf expansion after the 5th cycle of drought and at the end of the experiment leaf area was lowered by 17% compared to control (Figure 3(b)).

**Figure 4:**
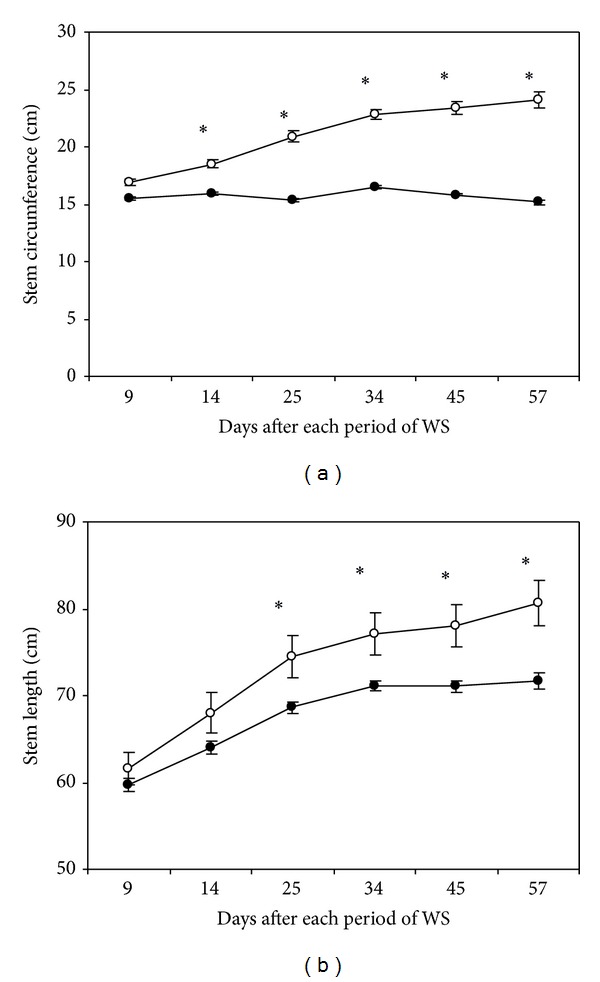
Pseudostem circumference (a) and length (b) determined in regularly irrigated (○) and water-stressed (●) banana plants. Data are means ± SE and each value was determined in three different plants with three replicates per treatment (*n* = 9). Data were compared for each date using the least significant difference (LSD) test. Significant differences at *P* ≤ 0.05 are represented by an asterisk (∗). WS: water stress.

Water stress arrested stem circumference expansion since the 1st cycle of drought whereas stem perimeter grew progressively (from 17 to 24 cm) in well irrigated plants (Figure 4(a)). At the end of the last drought cycle such decrease was 20% with respect to control. Moreover, irrigated plants showed a continuous increase of stem length (from 61 to 80 cm) during trial period (Figure 4(b)). However, drought detained completely stem elongation after the 4th cycle and diminution with respect to control was 11% at the end of the last cycle of drought.

### 3.3. Leaf Gas Exchange

**Figure 5:**
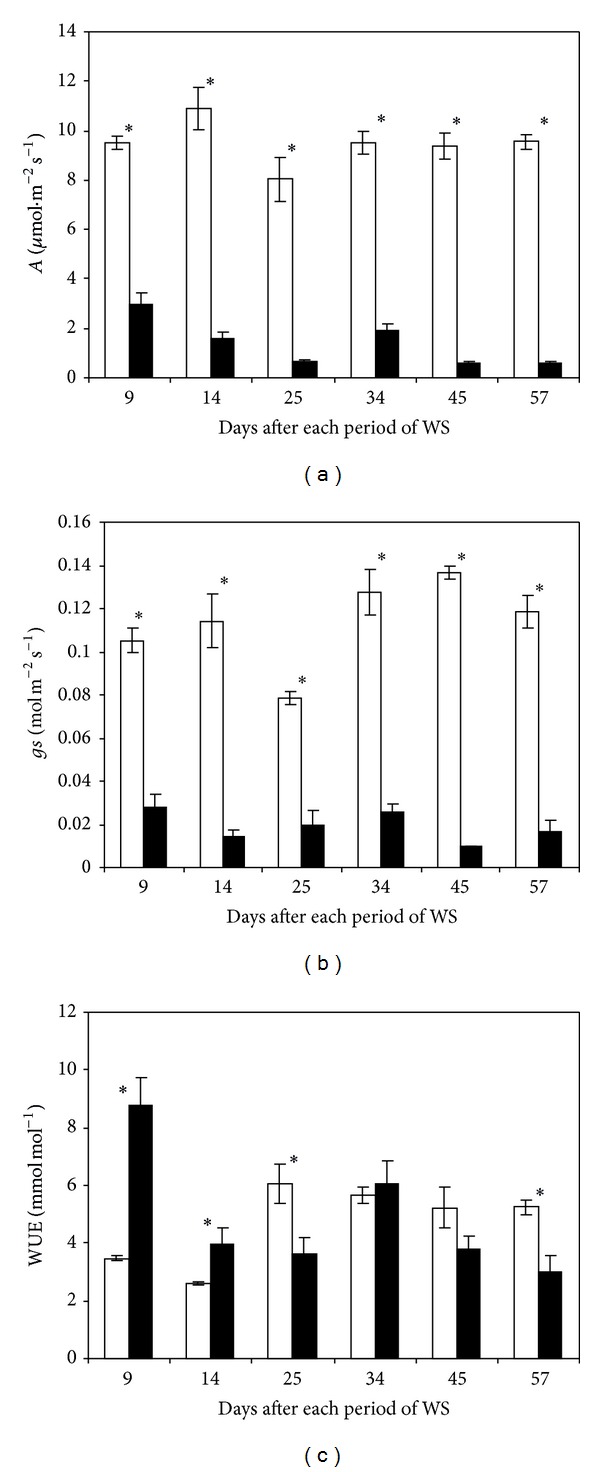
Photosynthetic rate (*A*), stomatal conductance (gs), and water use efficiency (WUE) in fully expanded leaves of banana plants. Treatments were regularly irrigated (□) and water-stressed (■) banana plants. Data are means ± SE and each value was determined in three different plants with three replicates per treatment (*n* = 9). Data were compared for each date using the least significant difference (LSD) test. Significant differences at *P* ≤ 0.05 are represented by an asterisk (∗). WS: water stress.

Photosynthetic rate (*A*) varied between 8 and 11 *μ*mol m^−2^ s^−1^ approximately during the whole trial period (Figure 5(a)). The various cycles of drought induced a severe decrease of net CO_2_ assimilation. In general such decreases varied between 68% (1st cycle) and 94% (last cycle) in comparison to control.

Stomatal conductance (gs) showed similar changes as *A* in control and water-stressed leaves (Figure 5(b)). gs values varied between 0.1 and 0.14 mol m^−2^ s^−1^ during the experiment in control plants; however, the imposed drought cycles reduced these values by between 73 and 92% in comparison to control.

The first two periods of drought increased water use efficiency (WUE) by about 60 and 34%, respectively, compared to control; however, afterwards drought decreased substantially WUE (42% compared to control at trial conclusion) except the 4th cycle which maintained similar WUE between control and water-stressed plants (Figure 5(c)).

### 3.4. Hormonal Changes

**Figure 6:**
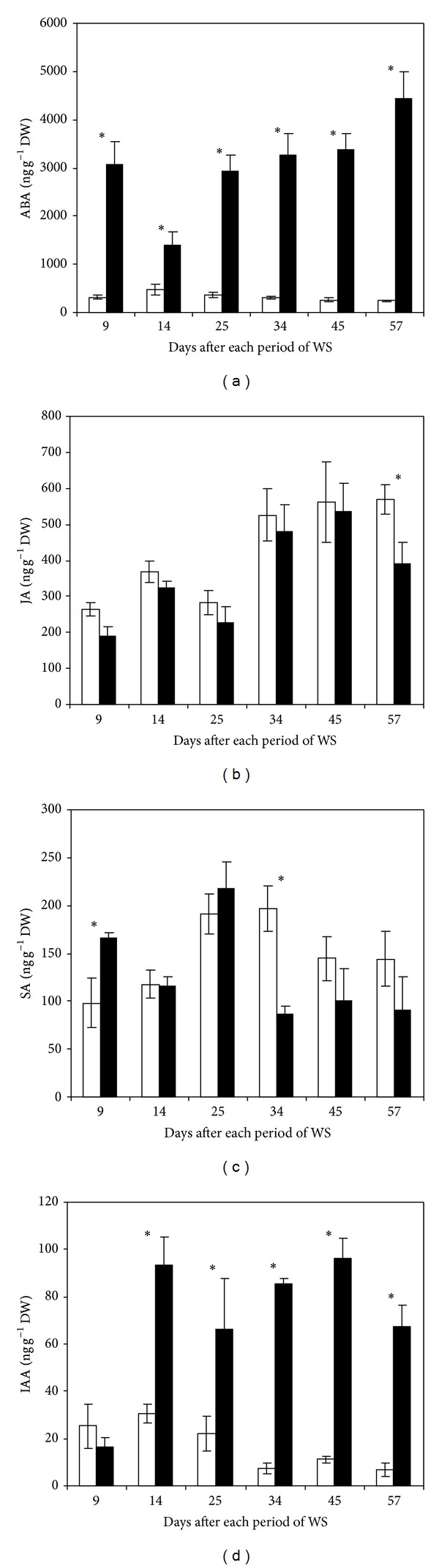
ABA (a), JA (b), SA (c), and IAA (d) concentrations in leaves from regularly irrigated (□) and water-stressed (■) banana plants. Data are means ± SE and each value was determined in three different plants with three replicates per treatment (*n* = 9). Data were compared for each date using the least significant difference (LSD) test. Significant differences at *P* ≤ 0.05 are represented by an asterisk (∗). WS: water stress.

Leaf ABA concentration oscillated between 245 and 472 ng g^−1^ DW during the experimental period (Figure 6(a)). The diverse periods of drought greatly increased ABA levels and such increases, for instance, were 10- and 18-fold compared to control after the 1st and the last period of drought, respectively.

Leaf JA accumulation in control plants showed similar range of concentration as ABA and varied between 263 and 570 ng g^−1^ DW throughout the trial period (Figure 6(b)). Nevertheless, the series of water stress periods did not change JA levels except at the end of trial when a reduction of JA was detected in comparison to control.

Foliar SA concentration fluctuated between 98 and 197 ng g^−1^ DW in well irrigated plants (Figure 6(c)); however, in dehydrated plants SA clearly increased after the 1st period of drought (40% with respect to control). Thereafter similar or lower concentrations of SA were detected in comparison with regularly hydrated plants.

In control plants, levels of IAA varied between 7 and 30 ng g^−1^ DW, being the high and low determinations at the beginning and the end of the experiment, respectively (Figure 6(d)). In contrast, except for the first one, all the remainder water stress periods highly increased foliar IAA concentration by about 66–91% compared to control.

### 3.5. Cinnamic and Ferulic Acids

**Figure 7:**
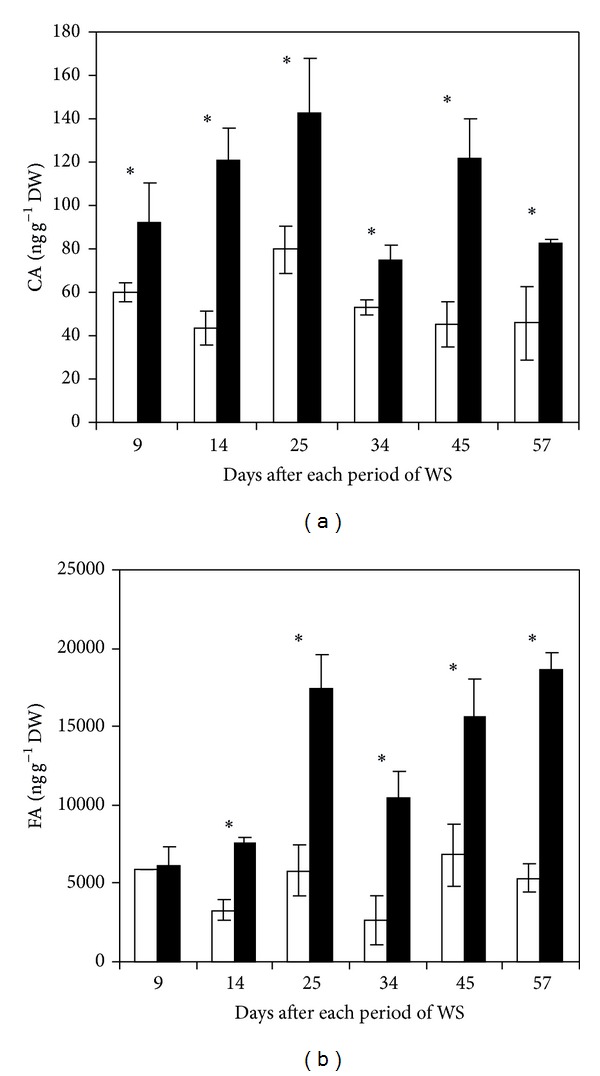
CA (a) and FA (b) concentrations in leaves from regularly irrigated (□) and water-stressed (■) banana plants. Data are means ± SE and each value was determined in three different plants with three replicates per treatment (*n* = 9). Data were compared for each date using the least significant difference (LSD) test. Significant differences at *P* ≤ 0.05 are represented by an asterisk (∗). WS: water stress.

Leaf cinnamic acid contents were 43–80 ng g^−1^ DW throughout all sampling dates in control plants (Figure 7(a)). Water removal increased cinnamic acid accumulation at each drought cycle conclusion. Minor (29%) and major (64%) increase with respect to control were obtained after the 4th and the 2nd period of drought, respectively.

High levels of ferulic acid (2680–5900 ng g^−1^ DW) were accumulated in banana leaves in well irrigated plants (Figure 7(b)). The effects of water stress on ferulic acid were similar to those on cinnamic acid, a general increase after each period of drought with the exception of the 1st one. Such increases reached a maximum of 74 and 71% compared to control at the 4th and 6th cycle of drought, respectively.

## 4. Discussion

Data presented here show that the accumulation of ABA is significant after periods of drought; however, such conditions did not alter the pattern of JA accumulation suggesting that its increase may likely occur at the onset of stress establishment as observed in previous experimental systems [9, 16]. The increase of ABA synthesis has been reported under short- or long-term water stress in several plant species and experimental systems [7–9, 47, 48]. In addition, the increase in ABA levels appears to correlate with plant adaptation to stress such as the decrease of the stomatal conductance, photosynthetic rate, and plant growth determined as stem height and circumference and leaf number and area. ABA has been considered one of the main signals that trigger plant acclimation under drought conditions and its biosynthesis is required for the induction and maintenance of stomatal closure during water stress [3, 6, 49]. Also, JA is involved in numerous physiological processes during plant development and in response to environmental stresses [15].

The increase of leaf IAA levels induced by drought suggests that this hormone can have a role on plant responses to dehydration. Although plant growth was significantly decreased, IAA may contribute at least to maintaining cell elongation activity at a low rate. Actually, the pattern of change of IAA under abiotic stress is not well established; however, it has been reported that an increase in its level occurs in roots under drought [18] and flooding [19].

SA seems to be involved in drought responses; however, that implication appears to be associated with the early stages of moisture depletion which would have a certain similitude with the transitory increase of JA [9, 16]. It has been reported that SA plays a role in regulating the drought response of plants and could be used as a potential growth regulator, for improving plant growth under water stress [26] and salinity [28].

Results also suggest that hydroxycinnamic acids may be involved in banana responses to water deficit. Thus, ferulic and cinnamic acids greatly increased in banana leaves at the end of each period of drought. In this process, ferulic and cinnamic acids could act as protectors of photosynthetic machinery and oxidative stress, respectively. This may explain the recovery of growth and net CO_2_ assimilation after each period of drought (data not shown). It has also been reported that ferulic acid may act as a light fitter limiting mesophyll penetration under drought conditions and can support drought adaption by downregulation of leaf growth [33]. Cinnamic acid, which is present in some plants in low amounts, also contributes to the overall blue-green fluorescence emission of leaves [32]. Drought stress accentuates banana leaf folding (data not shown) which reduces leaf area exposed to irradiation and water loss. Our data might suggest that this mechanism appears to be concomitant with the high accumulation of FA and CA as a leaf photoprotector. In conclusion, data suggest that most of the analysed metabolites might be involved in the regulation of plant responses to water stress. The increase of IAA concentration could be directed to avoid senescence in the survival leaves allowing them to maintain certain cell elongation rates. The accumulation of FA and CA could play key roles as photoprotectors leading to reducing irradiation area and water loss through leaf folding, reinforcing the effect of ABA on inducing stomatal closure. Data also suggest that SA and JA were not involved in water stress responses under consecutive periods of drought; however, their role might be limited to a transient and rapid increase at the onset of the first period of stress.
